# Seminal plasma metabolomics and lipidomics profiling to identify signatures of pituitary stalk interruption syndrome

**DOI:** 10.1186/s13023-022-02408-4

**Published:** 2022-07-15

**Authors:** Ye Guo, Xiaogang Li, Xi Wang, Haolong Li, Guoju Luo, Yongzhen Si, Xueyan Wu, Yongzhe Li

**Affiliations:** 1 Department of Clinical Laboratory, State Key Laboratory of Complex Severe and Rare Diseases, Peking Union Medical College Hospital, Chinese Academy of Medical Science and Peking Union Medical College, 1 Shuaifuyuan Road, Beijing, 100730 China; 2 Medical Science Research Center, State Key Laboratory of Complex Severe and Rare Diseases, Peking Union Medical College Hospital, Chinese Academy of Medical Science and Peking Union Medical College, Beijing, China; 3 National Health Commission Key Laboratory of Endocrinology (Peking Union Medical College Hospital), Department of Endocrinology, State Key Laboratory of Complex Severe and Rare Diseases, Peking Union Medical College Hospital, Chinese Academy of Medical Science and Peking Union Medical College, No. 1 Shuai Fuyuan, Dong Cheng District, Beijing, 100730 China

**Keywords:** Pituitary stalk interruption syndrome, Metabolomics, Lipidomics, Biomarker, Rare disease

## Abstract

**Background:**

Pituitary stalk interruption syndrome (PSIS) is a rare disease caused by congenital pituitary anatomical defects. The underlying mechanisms remain unclear, and the diagnosis is difficult. Here, integrated metabolomics and lipidomics profiling were conducted to study the pathogenesis of PSIS.

**Methods:**

Twenty-one patients with PSIS (BD group) and twenty-three healthy controls (HC group) were enrolled. Basal information and seminal plasma samples were collected. Untargeted metabolomics and lipidomics analyses were performed using ultraperformance liquid chromatography-quadrupole time-of-flight mass spectrometry (UPLC-QTOF-MS).

**Results:**

The metabolomics and lipidomics profiles of patients with PSIS changed. The prolactin signaling pathway and biosynthesis of amino acids were the main differentially modified metabolic pathways. The main differentially modified metabolites were triacylglycerols (TGs), phosphatidylethanolamine (PE), sphingomyelin (SM), ceramide (Cer) and phosphatidylcholines (PCs). Pregnenolones and L-saccharopine could achieve a diagnosis of PSIS.

**Conclusions:**

Pregnenolones and L-saccharopine are potential biomarkers for a PSIS diagnosis.

**Supplementary Information:**

The online version contains supplementary material available at 10.1186/s13023-022-02408-4.

## Introduction

Pituitary stalk interruption syndrome (PSIS) is a clinical syndrome characterized by the lack or thinning of the pituitary stalk, pituitary hypoplasia, and/or ectopic neurohypophysis. PSIS is a rare disease with an incidence of 1 in 200 000 [1]. Hormones secreted by the hypothalamus cannot be transported to the anterior and posterior pituitary through the pituitary portal system due to the interrupted pituitary stalk. Defects in growth and pubertal development are the main clinical manifestations. Hormone replacement therapy is required to promote sexual development, maintain sexual function and induce spermatogenesis [2, 3]. The diagnosis of PSIS is challenging, and magnetic resonance imaging (MRI) may be useful [4–6].

Metabolomics is commonly used to simultaneously analyze all metabolites in organisms. It helps identify the relationships between metabolites and pathological changes. Most of its research objects are small molecular substances with a molecular mass of less than 1000 Da [7]. Many biomarkers for diagnosis and monitoring therapy have been developed using this method. Previous studies of seminal plasma from infertile men show that metabolites, such as amino acids and fatty acids, are potential biomarkers [8, 8–10]. Therefore, we aimed to identify biomarkers of PSIS by metabolomics analysis, which may help improve the therapeutic effects on spermatogenesis. Here, integrated metabolomics and lipidomics analyses were performed to identify the specific metabolites associated with PSIS.

## Materials and methods

### Participants

In this study, 21 patients with PSIS (BD group) and 23 healthy controls (HC group) according to the 5th edition of the WHO Manual for the Laboratory Examination and Processing of Human Semen were recruited from Peking Union Medical College Hospital (PUMCH) from January 2020 to March 2021. The diagnostic criteria included (a) growth hormone deficiency and (b) a triad of an absent or thin pituitary stalk, pituitary hypoplasia, and/or ectopic neurohypophysis visible on magnetic resonance imaging (MRI). All patients and HCs enrolled were of Han Chinese ethnicity. The sample should be collected after a minimum of 2 days and a maximum of 7 days of sexual abstinence. If additional samples are required, the number of days of sexual abstinence should be as constant as possible at each visit.. All sex hormones were measured using an automated chemiluminescence immunoassay analyzer (Beckman Coulter UniCel DXI 800, Beckman Coulter; Brea, CA, USA). Sperm motility and concentration assessments were performed using computer-assisted sperm analysis systems (Suiplus SSA-II, Suiplus Software Co. Ltd.; Beijing, China). The microscope used with SSA-II was a Nikon 80i with a 20phase objective. The baseline information of all participants is listed in Table 1. Sample collection and laboratory parameters are described in Additional file 1: S1.

**Table 1:** Clinical characteristics of all participants

Parameter	PSIS patients (n = 21)	Healthy control (n = 23)	Normal reference ranges
Age at sample time (years)	24.6 ± 4.9	24.3 ± 5.7	–
Treatment duration(years)	5.3 ± 2.0	–	–
Malpresentation	21/21	Negative	Negative
Obesity	3/21	0/23	BMI ≥ 30
Overweight	8/21	4/23	BMI ≥ 25
Normal	8/21	17/23	BMI ≥ 18.5
Low-body weight	2/21	1/23	BMI < 18.5
Cryptorchidism	1/21	Negative	Negative
Testicular volume(ml)	5.9 ± 2.6	13.5 ± 3.1*	12–20
Azoospermia	7/21	Negative	Negative
Semen non-liquefaction	10/21	Negative	Negative
Semen condensed	9/21	Negative	Negative
Sperm count (million)	20.4 ± 29.4	229.4 ± 118.6*	≥ 39
Sperm concentration (million/mL)	8.7 ± 13.2	96.1 ± 55.2*	≥ 15
Sperm motility (%)	33.9 ± 28.1	69.7 ± 12.3*	≥ 40 (PR + NP)
Seminal fructose (Positive)	21/21	22/22	Positive
FSH(IU/L)	1.29 ± 1.47	6.43 ± 4.34*	1.27–19.26
LH(IU/L)	0.38 ± 0.59	3.59 ± 1.57*	1.24–8.62
T(ng/mL)	1.85 ± 1.03	8.4 ± 12.7*	1.75–7.81
PRL (ng/mL)	14.5 ± 8.6	12.1 ± 6.3	2.6–13.1
E2(pg/mL)	22 ± 13	24 ± 8	< 39
FT4(ng/dL)	0.75 ± 0.25	1.03 ± 0.56*	0.18–1.89
FT3(ng/dL)	2.53 ± 0.47	2.80 ± 0.71	1.80–4.10
IGF1(ng/mL)	101 ± 62	252 ± 76*	127–424

*FSH* follicle-stimulating hormone, *LH* luteinizing hormone, *T* testosterone, *E2* estradiol, *PRL* prolactin, *FT3* estradiol, *FT4* prolactin, *IGF1* insulin-like growth factor

*means *P* < 0.05

### Materials and instruments

Chromatographic grade ammonium fluoride, 2-propanol, ammonium acetate, formic acid, ammonium hydroxide, ammonium formate, acetonitrile, and methyl tert-butyl ether (MTBE) were obtained from Sigma Aldrich. Mass spectrometry grade methanol was purchased from Thermo Fisher. The Q-Exactive Plus Orbitrap LC–MS/MS System was from Thermo Scientific. AB Triple TOF 6600 was purchased from AB Sciex (Massachusetts, USA). The Nexera LC-30A liquid chromatography system was from SHIMADZU. An ACQUITY UPLC BEH Amide column (1.7 μm, 2.1 mm × 100 mm) and ACQUITY UPLC CSH C18 column (1.7 µm, 2.1 mm × 100 mm) were obtained from Waters Corporation (Milford, MA, USA).

### Sample preparation for metabolomics and lipidomics profiling

For metabolomics profiling, the seminal plasma sample was extracted by methanol/acetonitrile. In brief, precooled methanol/acetonitrile/water solution (2:2:1, v/v) was mixed with 10 μL of the sample and sonicated for 30 min. The samples were centrifuged at 14,000 × g for 20 min at 4 °C to obtain the supernatant. Then, it was dried in a vacuum centrifuge and redissolved in 100 μL acetonitrile/water (1:1, v/v). The samples were centrifuged at 14,000 × g for 15 min, and the supernatant was used for the analysis.

For lipidomics profiling, 240 μL precooled methanol, 200 μL water and 20 μL internal lipid standard mixture were homogenized with the seminal plasma sample. MTBE (800 μL) was added and sonicated for 20 min. Then, the samples were centrifuged at 14,000 × g for 15 min to obtain the supernatant. After drying under nitrogen, the samples were redissolved in 200 μL of 90% isopropanol/acetonitrile solution and centrifuged at 14,000 × g for 15 min. The final supernatant was used for lipidomics analysis. The whole process was performed under refrigeration.

### Quality control

Equal amounts of the supernatants from the seminal plasma samples were mixed to prepare the quality control (QC) sample. The QC sample was used to determine the instrument state and evaluate the system’s stability during the entire experiment.

### Mass spectrometry data acquisition and processing

A quadrupole time-of-flight 6600 (AB Sciex) was used for metabolomics data acquisition. Lipidomics data acquisition was performed using a Q-Exactive Plus Orbitrap LC–MS/MS System (Thermo Scientific). The gradients of both separations and the ESI source conditions are described in Supporting Information S2 and S3. Further information on both the separations and ESI source conditions is shown in Additional file 1: S2 and S3.

The metabolomics raw MS data were processed using ProteoWizard and XCMS software. The lipidomics raw MS data were processed using Lipid Search, including peak identification, peak extraction, and lipid identification (secondary identification). Further information is shown in Additional file 1: S4.

### Statistical analysis

Data with a normal distribution are expressed as the mean (SD). Statistical analysis was performed using the R package. Student’s t-test at the univariate level was used to measure the significance of the metabolites, and *p* values < 0.05 were considered significant. MetaboAnalyst 5.0 (Xia Lab @ McGill Sweden) was used to perform chemometrics, cluster and biomarker analyses.

## Results

### Clinical characteristics of the patients with PSIS

All of the patients with PSIS had a history of malpresentation (100%). They were 24.6 ± 4.9 years old. Combined gonadotropins (HCG/HMT) were administered for 18 ± 6 months to induce spermatogenesis.The patients did not take other medications such as psychotropic drugs, which may interfere with the results of metabolomics and lipidomics profiling [11]. All of them experienced erections and seminal fluid production. Nonliquefaction occurred in 10/21 (47.6%) samples, and condensed semen occurred in 9/21 (42.8%) samples. Patients with PSIS had a smaller testicular volume (PSIS Group 5.9 ± 2.6 ml vs. HC Group 13.5 ± 3.1 ml), lower sperm counts (PSIS Group 20.4 ± 29.4 × 10^6^ vs. HC Group 229.4 ± 118.6 × 10^6^), sperm concentrations (PSIS Group 8.7 ± 13.2 × 10^6^/mL vs. HC Group 96.1 ± 55.2 × 10^6^/mL) and sperm motility (PSIS Group 33.9 ± 28.1% vs. HC Group 69.7 ± 12.3%). Patients with PSIS had lower levels of luteinizing hormone (LH) (PSIS Group 0.38 ± 0.59 IU/L vs. HC Group 3.59 ± 1.57 IU/L), serum follicle-stimulating hormone (FSH) (PSIS Group 1.29 ± 1.47 IU/L vs. HC Group 6.43 ± 4.34 IU/L) and testosterone (T) (PSIS Group 1.85 ± 1.03 ng/mL vs. HC Group 8.4 ± 12.7 ng/mL) (Table 1).

### Metabolomics and lipidomic profiling of seminal plasma

**Fig. 1:**
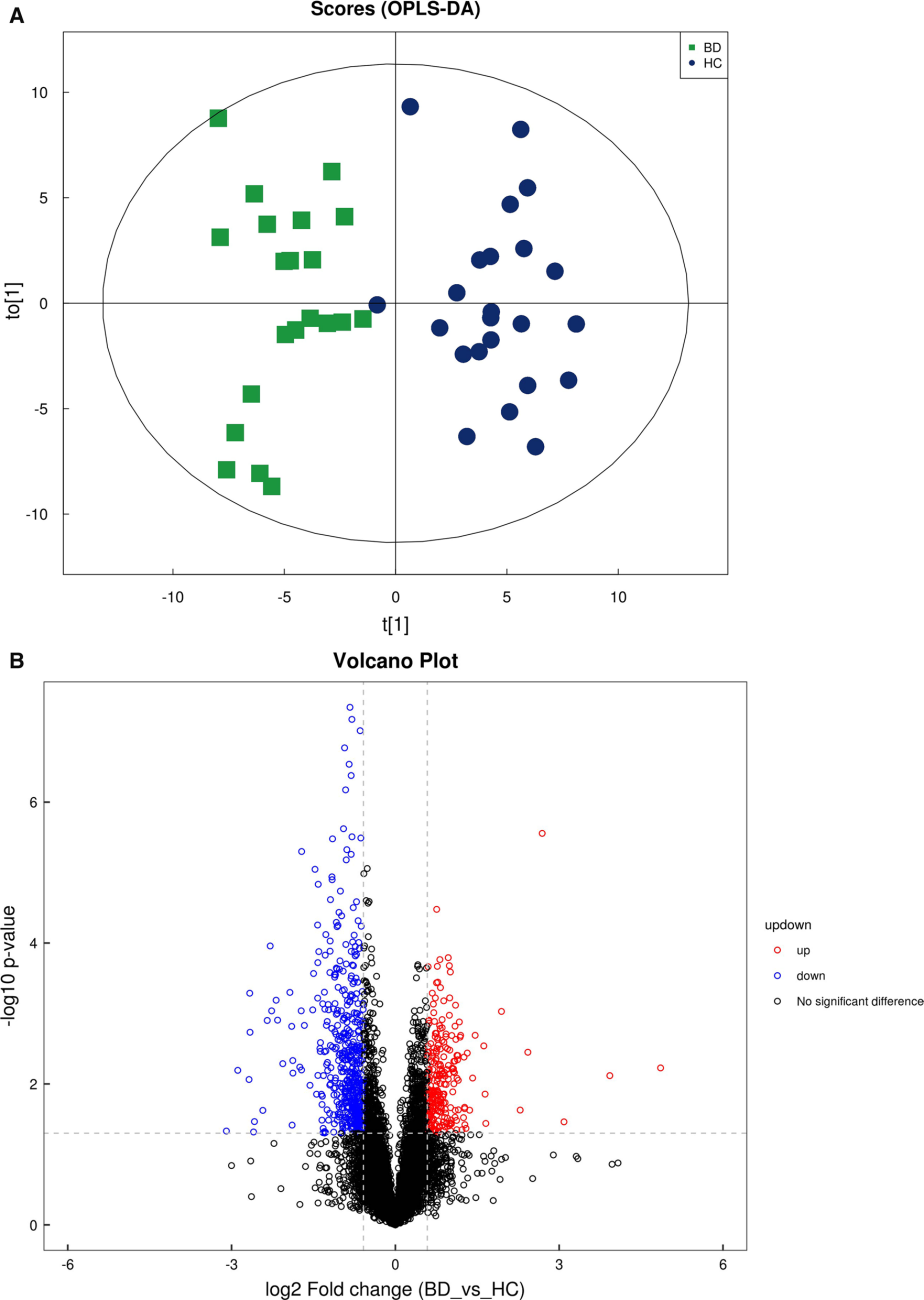
(**A**) OPLS-DA model of metabolomics in ESI + ionization mode. (**B**) Volcano plot of metabolomics in ESI + ionization mode

**Fig. 2:**
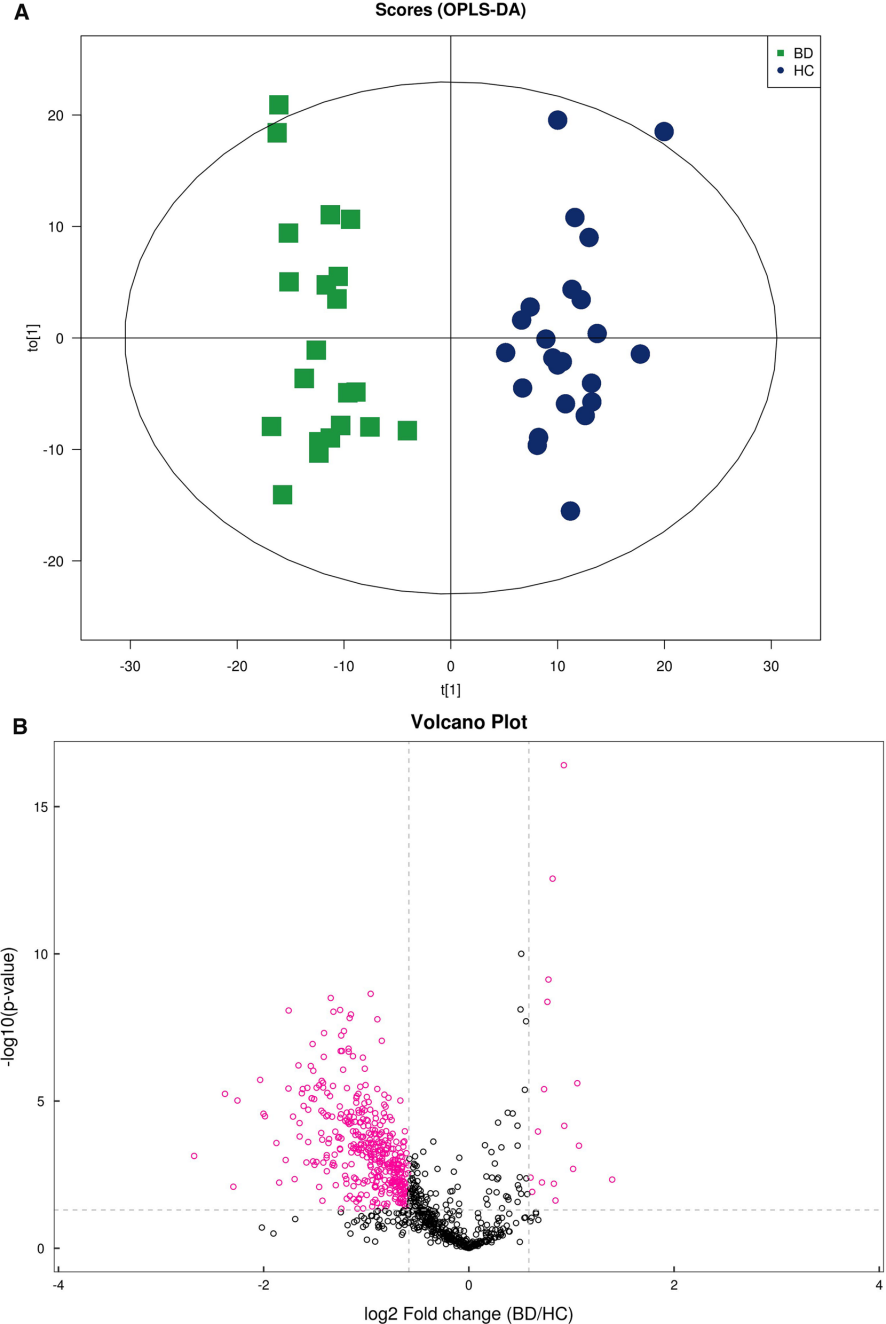
(**A**) OPLS-DA model of lipidomics for PSIS (BD group, green) vs. control (HC, blue). (**B**) Volcano plot of lipidomics for PSIS (BD group) vs. HC

Using a UPLC–MS approach, we analyzed the metabolomics and lipidomic profiles of patients with PSIS. To characterize the differences, orthogonal projections to latent structures-discriminant analysis (OPLS-DA) plots were generated (Fig. 1A, Fig. 2A). As shown in our OPLS-DA charts, the PSIS and HC groups were separated in both ion modes. Many metabolites were decreased in patients with PSIS, as the colored dots in the volcano plot indicate significantly different molecules (Figs. 1B, 2B). The criteria were set as variable importance in the projection (VIP) > 1 and *p* < 0.05 (Student’s t-test).

**Fig. 3:**
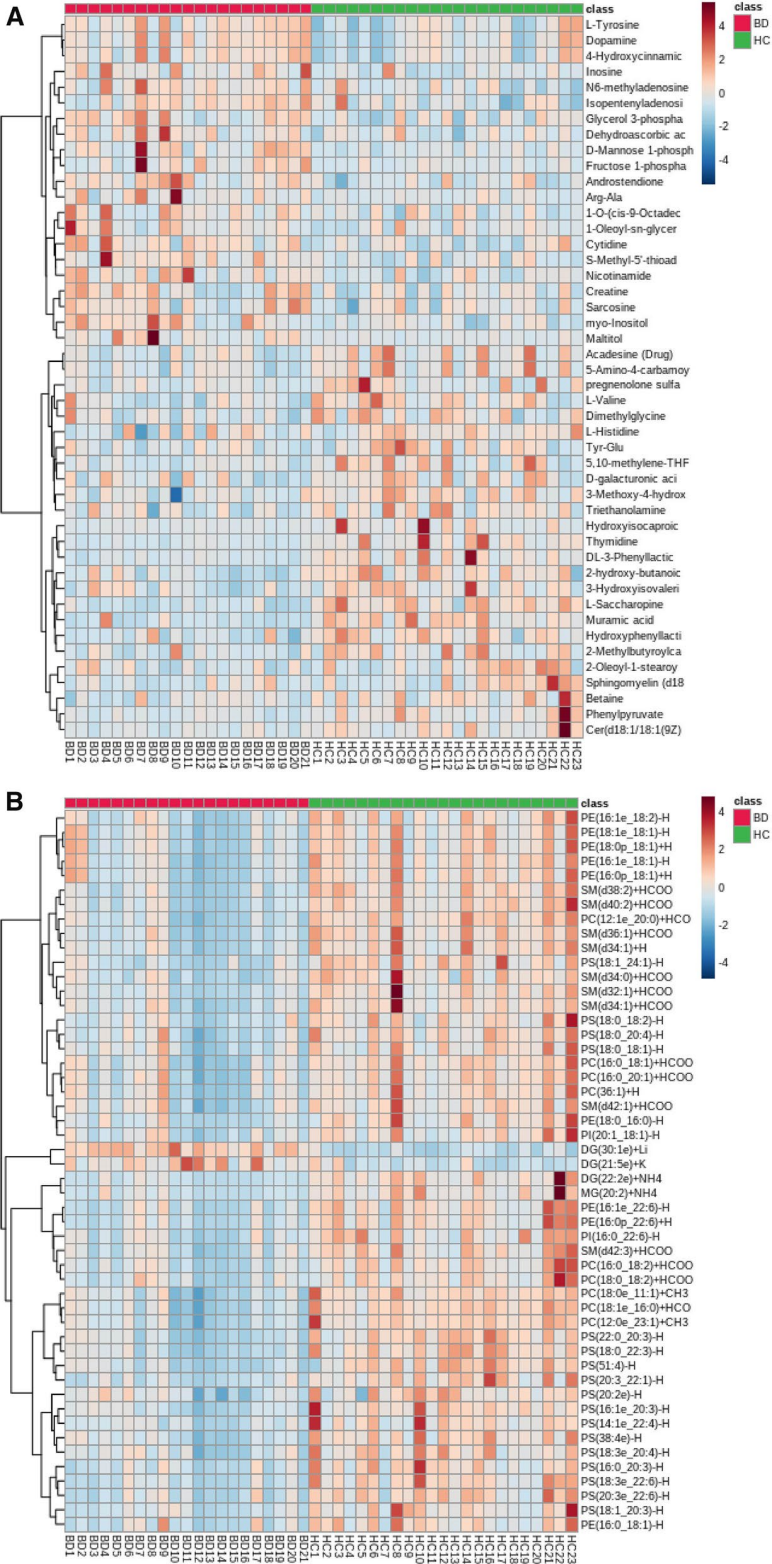
(**A**) Heatmap of the top differential features in metabolomics. (**B**) Heatmap of the top different features in lipidomics

**Fig. 4:**
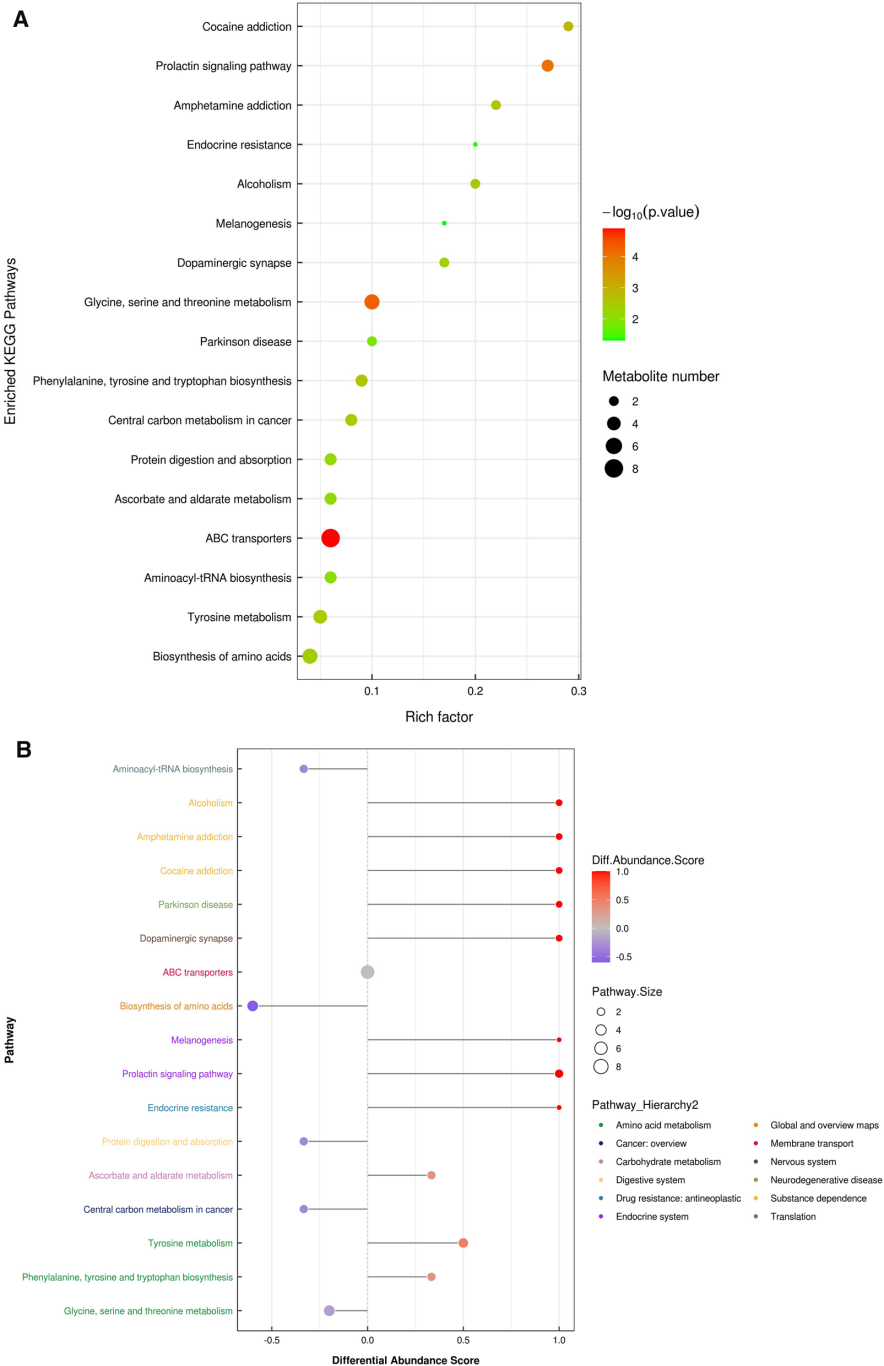
(**A**) KEGG enrichment analysis for the PSIS vs. HC groups. (**B**) Differential abundance score analysis for the PSIS vs. HC groups

**Fig. 5:**
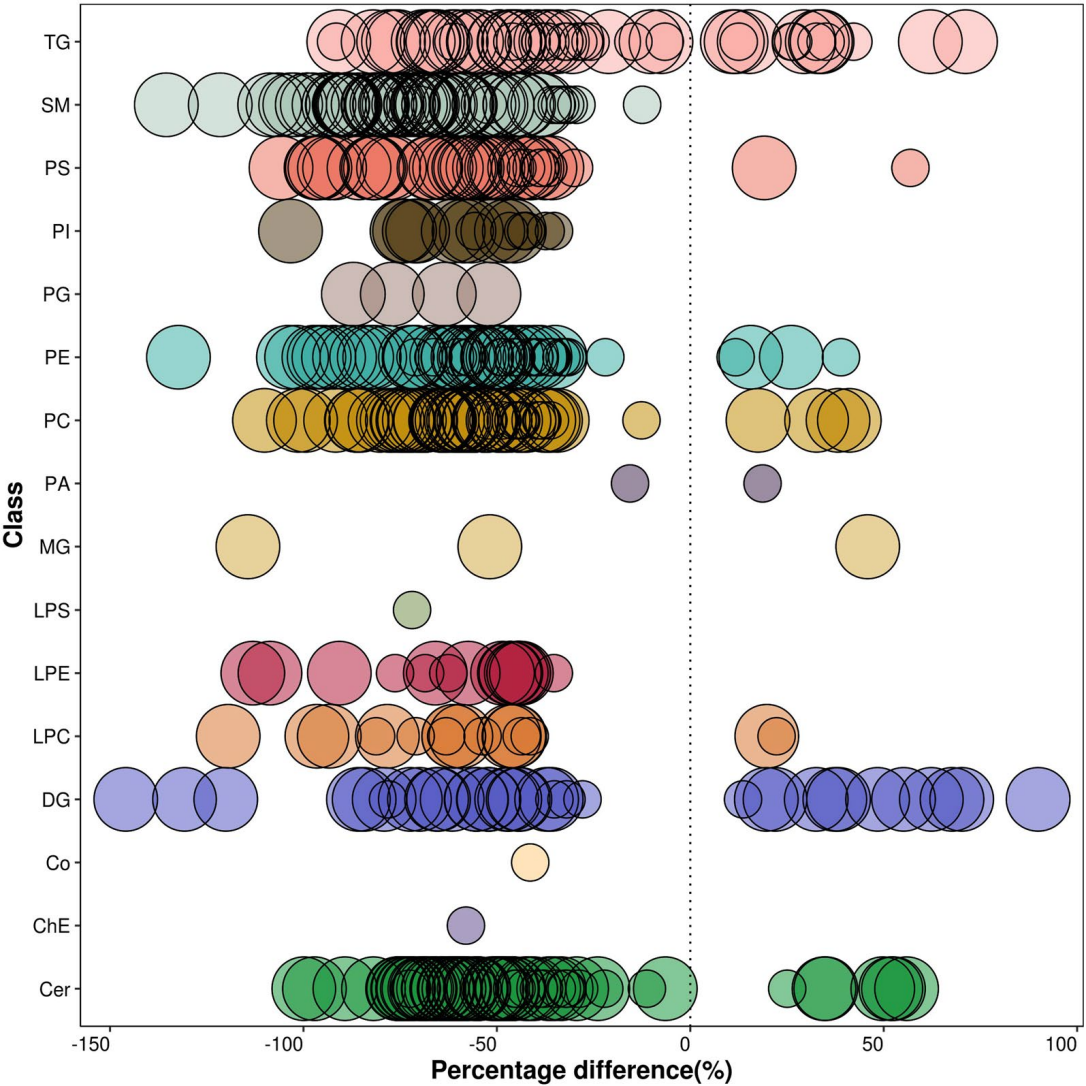
Lipid group bubble plot for the PSIS vs. HC groups

**Fig. 6:**
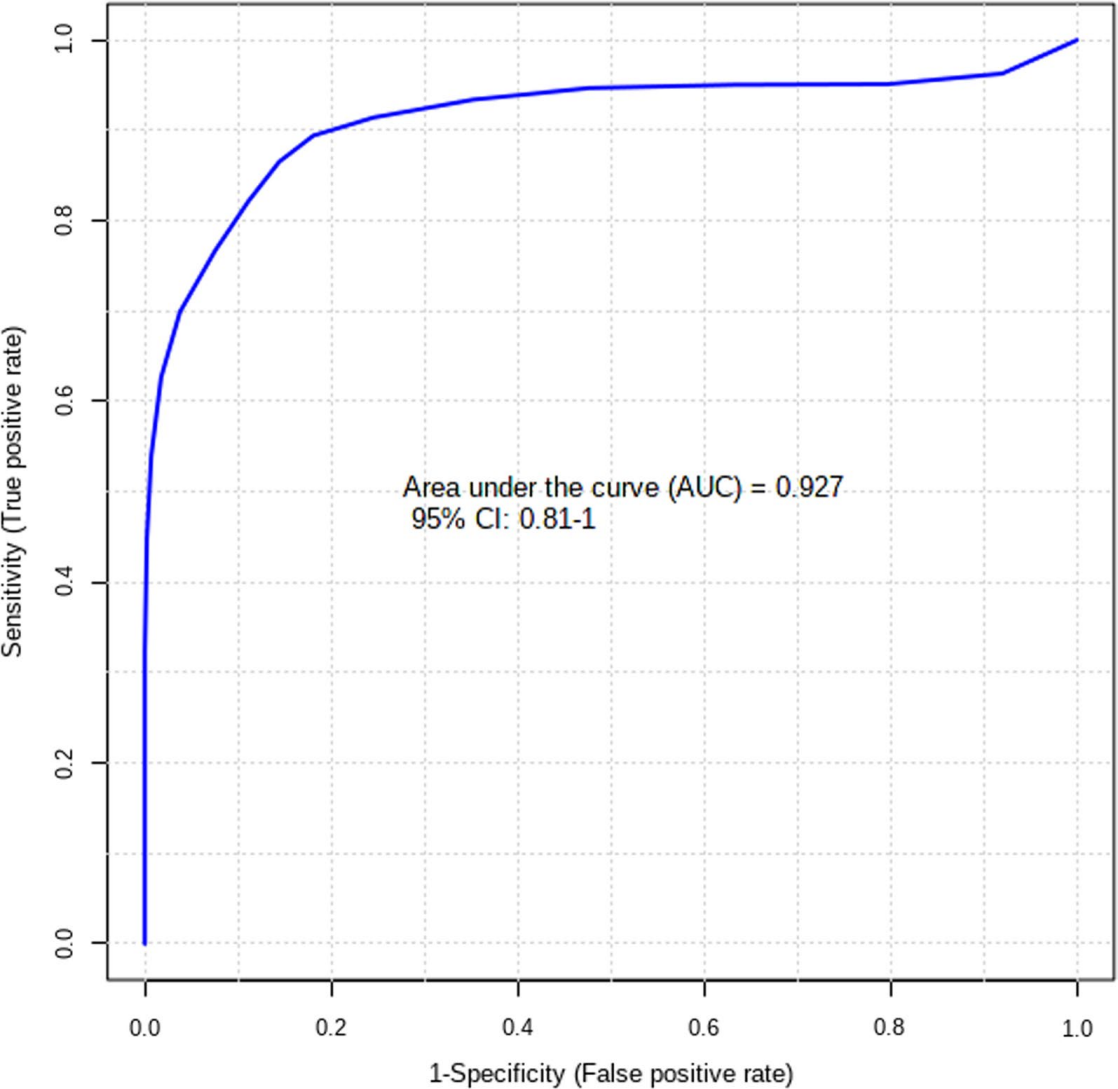
The ROC curve of the panel generated by combining pregnenolone sulfate and L-saccharopine

As Fig. 3A shows, in the metabolomics profiles, metabolites were significantly different among the different groups (Additional file 1: Table S1). Many lipid species, such as triacylglycerol (TG), sphingomyelin (SM), phosphatidylethanolamine (PE), phosphatidylcholines (PC), and ceramides (Cer), were decreased in the PSIS group (Fig. 3B, Additional file 1: Table S2). The KEGG pathway enrichment analysis showed that the prolactin signaling pathway and biosynthesis of amino acids were different in patients with PSIS (Fig. 4A). The size of the bubble indicates the P value, and the color indicates the significance of the enrichment degree. The overall changes in the pathway are shown in the differential abundance score (DAS) (Fig. 4B). The length represents the absolute value of the DA score, and the size indicates the number of metabolites. For lipid analysis, a bubble plot was used to display the significance of the differences and the classification information for different metabolites (Fig. 5). The size of the bubbles represents the significance of the differences (significant: 0.01 < *p* < 0.05; extremely significant: *p* < 0.01). As shown in Fig. 5, TG, SM, PE, PC and Cer were the main differential lipid molecules. The significantly different metabolites were identified with receiver operating characteristic (ROC) analysis. The diagnostic panel model was optimized by using multivariate exploratory ROC analysis. By combining PS and L-saccharopine, we achieved excellent differentiation of PSIS: PSIS vs. HC (AUC = 0.927, 95% Cl: 0.81–1) (Fig. 6).

## Discussion

The baseline characteristics are after combined gonadotropins administration. The patients did not receive testosterone therapy before HCG/HMT treatment. This untargeted metabolomics and lipidomics study demonstrated that the prolactin signaling pathway and biosynthesis of amino acids were different in patients with PSIS. TGs, SM, PE, Cer and PCs were decreased in lipid profile analysis of the seminal fluid from PSIS patients.

TG is the core of lipoprotein and serves as an energy source for most tissues. Previous studies have shown that some kinds of fatty acids are potential biomarkers of semen quality [12]. Diacylglycerol (DG, DAG) is formed through the hydrolysis of TG. In our data, most DGs decreased in patients with PSIS, while DG(32:1e), DG(30:1e), and DG(21:5e) increased.

PC is the main component of biofilms, and it is composed of a hydrophilic head and a hydrophobic tail [13, 14]. PC(12:1e_20:0), PC(16:0_18:1), PC(16:0_20:1), PC(16:0_18:2), PC(36:1), PC(18:0_18:2), PC(18:0e_11:1), PC(18:1e_16:0), and PC(12:0e_23:1) were decreased in patients with PSIS. The changed composition and saturation of PC may impair the function of the membranes of sperm [13–17, 17, 18]. Phosphatidylethanolamine (PE) is the major component of these membranes.

In our data, most kinds of lipids decreased, which revealed a poor nutritional microenvironment. PE(16:1e_18:2), PE(18:1e_18:1), PE(18:0p_18:1), PE(16:1e_18:1), PE(16:0p_18:1), PE(18:0_16:0), PE(16:1e_22:6), PE(16:0_22:6), and PE(16:0_18:1) were the significantly different metabolites (Fig. 3B). The decreased PE may lead to inflammatory response disorders and cause semen nonliquefaction (13/21) and semen viscosity (12/21). The findings of our study support the results of a previous study showing that phosphatidylethanolamine binding protein 4 is a potential proteomic marker for semen quality [19, 20].

Cer is a metabolite of SM and it is the main component of the phospholipid bilayer sphingomyelin. SM(d38:2), SM(d40:2), SM(d36:1), SM(d34:1), SM(d42:1), and SM(d42:3) were significantly decreased in the patients with PSIS. Ceramide can participate in a variety of cell signaling pathways, such as cell growth, differentiation, and apoptosis. In our data, most kinds of lipids were decreased, which revealed the poor nutritional microenvironment [21, 22]. Patients with PSIS had a smaller testicular volume (PSIS Group 5.9 ± 2.6 ml vs. HC Group 13.5 ± 3.1 ml), lower sperm counts (PSIS Group 20.4 ± 29.4 × 10^6^ vs. HC Group 229.4 ± 118.6 × 10^6^), sperm concentrations (PSIS Group 8.7 ± 13.2 × 10^6^/mL vs. HC Group 96.1 ± 55.2 × 10^6^/mL) and sperm motility (PSIS Group 33.9 ± 28.1% vs. HC Group 69.7 ± 12.3%), which may be associated with the barren spermatogenesis microenvironment.

PS is a natural steroid synthesized from pregnenolone (P5), and it can be converted into endogenous steroid hormones such as progesterone (P4) and estrogen (E2). Neuroactive steroids synthesized directly in the brain are independent of peripheral sources and they rapidly alter neuronal excitability by binding to their receptors [23]. PS is endogenously present in the brain and is synthesized by glial cells, which modulate neurotransmission in various systems through presynaptic and postsynaptic mechanisms [24]. In addition, it can also promote sleep quality and improve memory, and it has many effects, such as anti-depression and anti-anxiety activity [25].

A previous study showed that PS could enhance prolactin (PRL) synthesis and secretion by triggering the activation of PKA, PKC and MAPK signaling. This is consistent with our data showing that the prolactin signaling pathway was the main differential metabolic pathway (Fig. 4B). PRL is a protein hormone secreted by anterior pituitary gland eosinophils that can promote the development and growth of mammary glands and stimulate the production of luteinizing hormone receptors. In our study, the patients with PSIS had lower LH (0.37 ± 0.57 IU/L vs. HC of 3.59 ± 1.57 IU/L), FSH (1.19 ± 1.49 IU/L vs. HC of 6.43 ± 4.34 IU/L) and T (1.74 ± 1.01 ng/mL vs. HC of 8.4 ± 12.7 ng/mL), which may be associated with their low level of PS.

## Conclusions

This untargeted metabolomics and lipidomics profiling of seminal plasma showed that the prolactin signaling pathway and biosynthesis of amino acids were different in patients with PSIS, indicating the underlying mechanism of PSIS. Furthermore, most types of lipids, such as TG, PC, PE, SM and Cer, were decreased, revealing a poor nutritional microenvironment for spermatogenesis. Supplementation with these lipid metabolites may improve spermatogenesis, which needs to be verified in future studies.

## Supplementary Information

**Additional file 1:** Supporting information.

## Data Availability

All data supporting the conclusions of this research article are included in the manuscript.

## Abbreviations

PSIS: Pituitary stalk interruption syndrome
MRI: Magnetic resonance imaging
TG: Triacylglycerol
PE: Phosphatidylethanolamine
SM: Sphingomyelin
Cer: Ceramide
PC: Phosphatidylcholine
FSH: Follicle-stimulating hormone
LH: Luteinizing hormone
T: Testosterone
E2: Estradiol
PRL: Prolactin
FT3: Estradiol
FT4: Prolactin
IGF1: Insulin-like growth factor
PR: Progressive motility
NP: Non-progressive motility
