# Supplementary material for: Seminal plasma metabolomics and lipidomics profiling to identify signatures of pituitary stalk interruption syndrome
Source: Orphanet J Rare Dis. 2022 Jul 15;17:267. doi: 10.1186/s13023-022-02408-4 (PMC9287950; doi:10.1186/s13023-022-02408-4)
Supplement: Supplementary file 1 — Additional file 1: Supporting information. [file 13023_2022_2408_MOESM1_ESM.doc]

**Supporting information**

**Seminal plasma metabolomics and lipidomics profiling to identify biomarkers of** **pituitary stalk interruption syndrome**

Ye Guo ^a #^, Xiaogang Li ^a, b #^, Xi Wang ^c #^, Haolong Li ^a^, Songxin Yan ^a^, Jiangfeng Mao ^c^, Xueyan Wu^c*^, Yongzhe Li ^a *^

a Department of Clinical Laboratory, State Key Laboratory of Complex Severe and Rare Diseases, Peking Union Medical College Hospital, Chinese Academy of Medical Science and Peking Union Medical College, Beijing, China

b Medical Science Research Center, State Key Laboratory of Complex Severe and Rare Diseases, Peking Union Medical College Hospital, Chinese Academy of Medical Science and Peking Union Medical College, Beijing, China

c National Health Commission Key Laboratory of Endocrinology (Peking Union Medical College Hospital), Department of Endocrinology, State Key Laboratory of Complex Severe and Rare Diseases, Peking Union Medical College Hospital, Chinese Academy of Medical Science and Peking Union Medical College, Beijing, China

**S1 Sample collection**

**S2 Gradient** **elution procedure and ESI source conditions for metabolomic lipidomic profiling**

**S3 Gradient** **elution procedure and ESI source conditions for lipidomic profiling**

**S4 Data processing**

**S1 Sample collection**

The preparation before sampling, blood sample collection and specimen processing were conducted according to IFCC/C-RIDL protocols. Fasting blood samples were taken via venipuncture into Vacuette tubes containing procoagulant, and within 15-30 min after sample collection, the samples were centrifuged at 1200×g for 10 min.

A semen sample from each participant was obtained by means of masturbation and ejaculation directly into noncytotoxic sterile containers. Freshly collected semen was liquefied for 30-60 min at room temperature and processed within 1 hour of ejaculation for analysis of the sperm characteristics according to the criteria published by the WHO. The samples were centrifuged at 1200×g for 10 min and frozen at −80 °C to obtain 200 μL of seminal plasma.

**S2 Gradient** **elution procedure and ESI source conditions for metabolomic lipidomic profiling**

ACQUITY UPLC BEH Amide column (1.7 μm, 2.1 mm× 100 mm, Waters Corporation, Milford, MA, USA) was used for HILIC separation. The autosampler temperature was set as 4 °C, and the injection volume was set as 2 μL. The column temperature was 25 °C. The flow rate was set as 0.5 mL/min. Mobile phase A contained 25 mM ammonium acetate and 25 mM ammonium hydroxide in water. Mobile phase B consisted of acetonitrile.

A gradient elution procedure was used, mobile phase B was 85% for 1 min and linearly reduced to 65% in 11 min, then was reduced to 40% in 0.1 min and kept for 4 min, and then increased to 85% in 0.1 min, with a 5 min re-equilibration period employed. The ESI source conditions were set as follows: ion source gas1 as 60, ion source gas2 (Gas2) as 60, curtain gas (CUR) as 30, source temperature: 600 °C, ion spray voltage floating (ISVF) ±5500 V. In MS only acquisition, the instrument was set to acquire over the m/z range 60-1000 Da, and the accumulation time for TOF MS scan was set at 0.20 s/spectra. In auto MS/MS acquisition, the instrument was set to acquire over the m/z range 25-1000 Da, and the accumulation time for product ion scan was set at 0.05 s/spectra. The product ion scan is acquired using information dependent acquisition (IDA) with high sensitivity mode selected. The parameters were set as follows: the collision energy (CE) was fixed at 35 V with ± 15 eV; declustering potential (DP), 60 V (+) and −60 V (−); exclude isotopes within 4 Da, candidate ions to monitor per cycle: 10.

**S3Gradient** **elution procedure and ESI source conditions for lipidomic profiling**

Lipidomics analyses were performed using a UHPLC (Nexera LC-30A, SHIMADZU) coupled to a Q-Exactive Plus Orbitrap LC-MS/MS System (Thermo Scientific). CSH C18 column (1.7 µm, 2.1 mm× 100 mm, Waters) was used for reverse phase chromatography separation. Solvent A: acetonitrile–water (6:4, v/v) with 0.1% formic acid and 0.1mm ammonium formate; solvent B: acetonitrile–isopropanol (1:9, v/v) with 0.1% formic acid and 0.1mm ammonium formate.

A gradient elution procedure was used, the initial mobile phase was 30% solvent B at a flow rate of 300 μL/min. It was held for 2 min, and then linearly increased to 100% solvent B in 23 min, followed by equilibrating at 5% solvent B for 10 min.Mass spectra was acquired by Q-Exactive Plus in positive and negative mode, respectively. ESI parameters were optimized and preset for all measurements as follows: Source temperature, 300 °C; Capillary Temp, 350 °C, the ion spray voltage was set at 3000V,S-Lens RF Level was set at 50% and the scan range of the instruments was set at m/z 200–1800.

**S4 Data processing**

The metabolomics raw MS data was processed using ProteoWizard. Peak picking and peak grouping were performed using XCMS software. We identified the metabolites by comparing MS/MS spectra and accuracy m/z value (<25 ppm) with an in-house database established with available authentic standards.

For lipidomics analysis, lipid Search was used to perform peak identification, peak extraction, and lipid identification (secondary identification). Lipid Search is a search engine whose database contains more than 30 lipid classes and more than 1,500,000 fragment ions. The main parameters are: precursor tolerance: 5 ppm, product tolerance: 5 ppm, product ion threshold: 5%.
